# Microplastic exposure in the lungs of young children and its associations with allergic rhinitis: A cross-sectional study in China

**DOI:** 10.1016/j.eehl.2025.100193

**Published:** 2025-10-24

**Authors:** Huimin Li, Jingli Yang, Lili Zhong, Gary W.K. Wong, Han Huang, Yinze Xu, Wendi Ma, Xuelin Lv, Li Peng, Dan Liu, Niguang Xiao, Shuhui Yin, Qiong Wang, Xiuqin Feng, Aimin Yang, Jingjing Zhang

**Affiliations:** a Department of Epidemiology and Statistics, School of Public Health, Hunan Normal University, Changsha 410013, China; b West China Biomedical Big Data Center, West China Hospital, Sichuan University, Chengdu 610041, China; c Hunan Provincial Key Laboratory of Pediatric Respirology, Pediatric Medical Center, Hunan Provincial People's Hospital (the First Affiliated Hospital of Hunan Normal University), Changsha 410005, China; d Department of Paediatrics, Prince of Wales Hospital, The Chinese University of Hong Kong, Hong Kong, China; e Hunan Maternal and Child Health Hospital, Changsha 410008, China; f China CDC Key Laboratory of Environment and Population Health, National Institute of Environmental Health, Chinese Center for Disease Control and Prevention (CDC), Beijing 100021, China; g Department of Medicine and Therapeutics, The Chinese University of Hong Kong, Prince of Wales Hospital, Hong Kong, China; h Li Ka Shing Institute of Health Sciences, The Chinese University of Hong Kong, Prince of Wales Hospital, Hong Kong, China

**Keywords:** Microplastic, Pyrolysis-gas chromatography/mass spectrometry, PA66, Allergic rhinitis, Children

## Abstract

Exposure to microplastics (MPs) has emerged as a potential threat to chronic respiratory health. However, the association between MPs exposure and allergic rhinitis (AR) in children remains unclear. We evaluated the association between MP exposure and the prevalence of AR in children. We measured 11 types of MPs in bronchoalveolar lavage fluid (BALF) collected from 207 children aged 1–16 years using pyrolysis-gas chromatography/mass spectrometry (Py-GC/MS) in 2023. Logistic regression models were employed to evaluate the association between MP concentration and prevalence of AR. Polyamide 66 (PA66), polyethylene (PE), polyvinyl chloride (PVC), and polystyrene (PS) were the predominant types detected, with median concentrations of 2.33, 0.45, 0.38, and 0.08 ​μg/mL in BALF, respectively. Higher concentrations of PA66 were associated with an increased prevalence of AR in all children, with odds ratios (ORs) of 3.00 (95% CI: 1.23, 7.34) after adjusting for potential confounders, indicating a statistically significant association (α ​< ​0.05). Higher concentrations of total MP exposure (*P*_overall_ ​= ​0.012, *P*_nonlinear_ ​= ​0.310) and PA66 exposure (*P*_overall_ ​= ​0.012, *P*_nonlinear_ ​= ​0.951) were significantly associated with the prevalence of AR in children aged ≤6 years but not in those aged >6 years. Our findings suggest that exposure to MPs, particularly PA66, may be associated with a higher risk of AR in younger children. Further large-scale, community-based pediatric cohort studies are needed to elucidate the underlying mechanisms.

## Highlights

• First study to link pediatric microplastics (MPs) exposure with allergic rhinitis (AR).
• PA66, PE, PVC, and PS were the predominant MPs in pediatric BALF.
• BALF MPs, particularly PA66, showed a linear association with the prevalence of AR.
• Associations between MPs and AR were mainly observed in children aged ≤6 years.

## Graphical abstract

**Figure:**
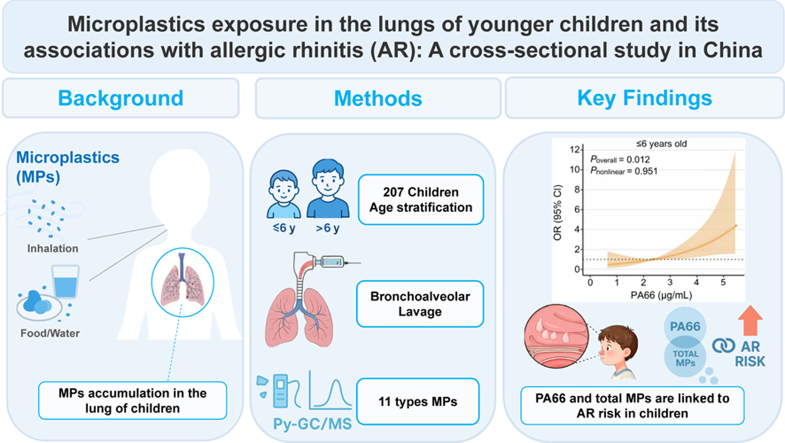

## 1 Introduction

Microplastics (MPs) are defined as solid plastic particles smaller than 5 ​mm [1]. Due to their small particle size and light weight, MPs are persistent environmental pollutants found across various environmental media [2]. Human exposure to MPs occurs through ingestion of food and water, inhalation of air, and dermal contact [3,4]. Zhang et al. identified the possible routes for MPs to enter and accumulate in the human body. They estimated that the human burden of MPs occurs via ingestion of drinking water and salt, and inhalation of air at (0–7.3) ​× ​10^4^, (0–4.7) ​× ​10^3^, and (0–3.0) ​× ​10^7^ items/(person·year), respectively, and that the amount of MPs inhaled through air was significantly higher than that ingested through other exposure routes [5]. Currently, MPs have been detected in lung tissue and bronchoalveolar lavage fluid (BALF), suggesting that the lungs are a primary target organ for MPs [2,6]. Therefore, the potential threat to respiratory health from long-term environmental MP exposure cannot be ignored, especially for chronic respiratory diseases such as asthma, allergic rhinitis (AR), and chronic obstructive pulmonary disease (COPD). Worryingly, the chronic respiratory effects of MP exposure in vulnerable populations, such as children, remain poorly understood.

Children are at a higher risk of exposure to environmental pollutants than adults due to their unique behavioral patterns. For instance, toys, infant bottles, and childcare products commonly contain polymers such as polyethylene (PE), polyvinyl chloride (PVC), and polyamide 66 (PA66), making children a key population at risk for MP exposure [6,7]. The concentration of MPs in children's BALF is 4.7 times higher than that in adults, particularly in newborns and young children, due to frequent crawling and tumbling indoors [[7], [8], [9]]. Simultaneously, these behavioral habits increase children's exposure to various allergens [10]. In addition, children's immune systems are not fully developed, and their resistance to external allergens is weak, which makes them more likely to develop chronic respiratory diseases [11]. Therefore, exploring the association between MP exposure and chronic respiratory diseases in children of different ages is significant.

AR is a leading chronic respiratory disease in children. In China, the prevalence of AR increased from 8.39% in 2012–2015 to 19.87% in 2016–2022, representing a 2.37-fold rise over the decade [12]. Among children under 6 years of age, the prevalence can reach up to 40% [13]. The rising prevalence of AR reflects the combined effects of genetic predisposition and environmental exposures, with particular concern about the role of novel environmental pollutants [14,15]. MP exposure has been shown to be elevated in AR patients, with BALF concentrations 1.3-fold higher than in healthy controls [16]. Increased absorption of MPs can induce oxidative stress, exacerbate airway inflammation, and affect multiple organ systems, thereby contributing to risks of non-communicable diseases, including cancer and chronic lung disease [17,18]. Animal studies further support that MP exposure has been linked to the onset and progression of allergic disease, with studies demonstrating heightened airway reactivity in mice [19,20]. In particular, co-exposure to polystyrene (PS) and the plasticizer DEHP significantly aggravated Th2 inflammation in allergic asthma via the TRPA1–p38 MAPK signaling axis. Beyond direct airway effects, MP derivatives also disrupt the gut microbiota, reducing short-chain fatty acids while increasing tryptophan metabolites, thereby promoting Th2-mediated pneumonia through the gut–lung axis [21,22]. However, the majority of existing research is limited to animal experiments, resulting in scarce and incomplete human evidence. Emerging data further suggest that micro- and nanoplastics can enter systemic circulation, trigger low-grade inflammation, and cause metabolic dysregulation—mechanisms that may impair immunity and heighten susceptibility to allergic diseases, including AR [[23], [24], [25]]. Taken together, these findings underscore the need for human-based investigations of the association between MP exposure and risk of pediatric allergic disease. To address this critical gap, our study evaluated the association between MP exposure and AR in a homogeneous cohort of children with *Mycoplasma pneumoniae* pneumonia (MPP).

We collected samples during a period of high MPP occurrence, when children with severe MPP required alveolar lavage. This study focused on the unique group of children with *Mycoplasma pneumoniae* infection, as their respiratory systems are in an acute inflammatory state and their bronchial epithelial barrier function is compromised, which may make them more susceptible to MP exposure [26]. The Global Initiative for Asthma (GINA) guidelines, which state that a child's asthma phenotype typically stabilizes by age 6, served as the basis for this stratification. Epidemiological studies of AR have demonstrated that children's symptomatic features and immunological response patterns tend to stabilize after age 6 [27,28]. Therefore, we investigated the association between exposure to different types of MPs and AR risk in children with MPP. The study was reported according to the Strengthening the Reporting of Observational Studies in Epidemiology (STROBE) guidelines [29].

## 2 Materials and methods

### 2.1 Study population

This is a cross-sectional study that included 207 children diagnosed with MPP who were admitted to Hunan Provincial People's Hospital, China, between October 2023 and December 2023. Participants were stratified by age, using 6 years as the cutoff, with 100 children aged ≤6 years and 107 children aged >6 years. This study was approved by the Ethics Committee of Hunan Provincial People's Hospital (approval number: [2024]-318).

The following inclusion criteria were applied: Pediatricians at Hunan Provincial People's Hospital diagnosed patients with MPP based on the guidelines provided in the Guidelines for the Diagnosis and Treatment of *Mycoplasma Pneumoniae* Pneumonia in Children (2023 Edition) [26]. A senior pediatric respiratory specialist verified the final diagnoses. Exclusion criteria included: (i) hospitalized children with pneumonia not related to MPP; (ii) BALF samples with less than 5 ​mL in volume; (iii) co-occurring respiratory, cardiovascular, or immunodeficiency conditions; and (iv) refusal to provide informed consent for participation in the study. In total, BALF samples were collected from 207 children aged between 1 and 16, along with clinical and demographic information.

### 2.2 Sample collection and quality control

All protocols involving human subjects strictly adhered to the Declaration of Helsinki. Throat swabs were collected from patients for quantitative polymerase chain reaction (qPCR) testing for MPP, after obtaining informed consent from participants and their families. When clinically indicated, children with MPP underwent bronchoalveolar lavage on the second day of hospitalization, following the Clinical Practice Guidelines for Bronchoalveolar Lavage in Chinese Children (2024 Edition) and the professional opinion of skilled medical professionals. A senior doctor with expertise in pediatric respiratory care performed all procedures consistently. The specific process for collecting BALF samples was described in Text S1.

Strict quality control protocols were implemented at all stages of sample collection, storage, processing, and analysis to prevent contamination from environmental plastics and ensure the accuracy of the results. To assess and correct for potential microplastic contamination introduced during sampling and pretreatment, both sample blanks and procedural blanks were included and processed using the same digestion and concentration procedures as the actual samples. Sample blanks consisted of sterile saline stored in glass bottles, injected through the bronchoscope, and withdrawn using a syringe to simulate the actual sampling process. Procedural blanks did not contain any biological material but included all reagents and equipment and were processed in the same manner as the samples. Measured MP concentrations in BALF samples were corrected by subtracting the background values detected in these blanks. The detailed results of both types of blanks are provided in Table S2.

In addition, to further minimize the risk of contamination during laboratory procedures, the use of plastic materials was avoided whenever possible. All work surfaces were covered with clean aluminum foil, and all experimental operations were conducted within a fume hood. Laboratory personnel wore only natural fiber clothing, clean cotton lab coats, and powder-free nitrile gloves. Sample collection and BALF procedures were performed in rooms that met operating-room cleanliness standards to minimize airborne microplastic contamination. All reagents used in the experiments, including deionized water and 75% ethanol, were triple-filtered through 0.45 ​μm polytetrafluoroethylene (PTFE) membranes. All glassware was rinsed three times with both filtered deionized water and ethanol. Reusable instruments were thoroughly cleaned prior to each experiment to maintain a clean laboratory environment and eliminate potential background contamination.

### 2.3 Pyrolysis-gas chromatography/mass spectrometry (Py-GC/MS)

BALF samples were transported to our laboratory for further analysis. Sample preparation followed an optimized acid digestion protocol, as previously cited in the literature, to remove contaminants [30]. Subsequently, we used a disposable glass Pasteur pipette to carefully transfer approximately 0.1 ​g of the concentrated sample into a Py-GC/MS crucible. The quality of the transferred liquid was recorded. The samples were then dried in an oven at 60 ​°C for 12 ​h until completely dehydrated. These prepared samples were then analyzed using a Py-GC/MS system, following standard analytical procedures for the detection and characterization of MPs. Py-GC/MS measurements are based on the thermal degradation of the polymer material.

In this study, pyrolysis (Py-3030D, Frontier), gas chromatography (GC 2030, Shimadzu), and mass spectrometry (MS-QP 2020NX, Shimadzu) were employed to detect possible plastic particles in the samples [31]. The SCAN pattern was chosen to identify and quantify the polymer of the target particles. The Py-GC/MS calibration curves for polymers are detailed in Table S1. The limit of detection (LOD) and limit of quantification (LOQ) were three and ten times the baseline noise, respectively (Table S1). The recovery test was conducted by adding known concentrations of MP solutions to three subsamples of BALF. The recovery rates for 11 types of MPs are listed in Table S3. This test was designed to evaluate the accuracy and efficiency of the analytical method in recovering MPs from complex biological samples such as BALF.

### 2.4 Scanning electron microscopy (SEM)

Representative aliquots of untreated BALF from the same batch used for Py-GC/MS analysis were separated into different portions. These portions were allocated to laser-direct infrared (LD-IR) imaging and SEM prior to Py-GC/MS to enable both morphological and chemical characterization. To preserve native particle integrity, these aliquots underwent no thermal treatment. Two-dimensional morphology and qualitative infrared spectra of particle spots were first obtained by LD-IR imaging. The same glass slides were subsequently sputter-coated with gold for SEM examination and were viewed at low accelerating voltages over a range of magnifications. Particle spots were manually relocated on the original glass carriers and imaged by SEM, guided by LD-IR-derived size and shape coordinates.

### 2.5 AR outcomes

AR outcomes were assessed using the International Study of Asthma and Allergies in Childhood (ISAAC) questionnaire [32]. We collected data on self-reported lifetime AR and doctor-diagnosed AR. Participants (or their guardians) were asked the question: “Has your child ever been diagnosed with AR by a doctor?”

### 2.6 Covariates

The following covariates were selected: (1) child characteristics, including sex (boy or girl), age, body mass index (BMI), vaginal delivery (yes or no), preterm delivery (yes or no), feeding pattern (breastfeeding, mixed feeding, and formula feeding), and additional vitamin C supplementation (yes or no); (2) characteristics of relatives, including parental education level (high school or below, bachelor's or junior college, master's degree or above); (3) family environment characteristics, passive smoking (yes or no), and whether the home has been renovated in the past year (yes or no).

### 2.7 Statistical analysis

Descriptive statistics were used to describe the basic characteristics of participants stratified by AR status. Data were expressed as mean ​± ​SD, medians with interquartile ranges (25th, 75th percentiles), or counts with proportions. Between-group differences were compared using appropriate tests, such as Student's *t*-test or Wilcoxon rank-sum test for continuous variables, and the χ^2^ test or Fisher's exact test for categorical variables. Spearman's rank correlation analysis was applied to evaluate the associations between MP concentrations. For MP values below the LOD, these values were replaced with the LOD divided by the square root of 2 in a lognormal distribution.

MP concentrations were analyzed both as a categorical variable (dichotomized at the median level, with specific cut-off values provided in Table S4) and as a continuous variable (log_10_-transformed to reduce skewness). A logistic regression model was used to evaluate the association between MP concentrations and prevalence of AR, expressed as odds ratios (ORs) with 95% confidence intervals (CIs). To further assess the dose–response associations, we modeled the MP concentrations as a continuous variable using restricted cubic spline regression with three knots placed at the 25th, 50th, and 75th percentiles of the MP distributions. Three models were specified: Model 1, unadjusted; Model 2, adjusted for age, sex, BMI, delivery mode, preterm birth, and parental education level; and Model 3, further adjusted for feeding pattern, passive smoke exposure, home decoration in the past year, vitamin C supplementation, and severity of MPP.

Subgroup analyses examined age-related heterogeneity by stratifying participants into those ≤6 years and those >6 years. Sensitivity analyses assessed robustness by excluding disease severity and adjusting for co-occurring MP polymers. To mitigate potential Type I error from multiple comparisons across polymers and subgroups, the Bonferroni correction was applied to the primary categorical analyses.

All analyses were conducted using R software (version 4.3.3; R Foundation for Statistical Computing, Vienna, Austria). Two-tailed tests were employed, and statistical significance was defined as *P* ​< ​0.05.

## 3 Results

### 3.1 Characteristics of the study population

Table 1 shows the basic characteristics of the 207 enrolled children, stratified by AR status. Of these, 98 (47.3%) were boys and 67 (32.4%) were diagnosed with AR. The mean age was 6.85 years and differed significantly between the two groups. In total, 107 children were >6 years and 100 were ≤6 years. The mean BMI was 15.89 ​kg/m^2^. Among all children, 118 (57.0%) were delivered vaginally, 8 (3.9%) were preterm, 113 (54.6%) were breastfed, 21 (10.1%) received vitamin C supplementation, and 70 (33.8%) were exposed to passive smoke. Most parents had a bachelor or junior college education, with 138 cases (66.7%), and 58 (28.0%) had a home renovation within a year. A total of 191 (92.3%) children developed severe pneumonia.

**Table 1:** Basic characteristics of the participants in this study (n ​= ​207).

Characteristics	Overall	Non-AR	AR	*P* value
No. of participants	207	140	67	
Boy, n (%)	98 (47.3)	63 (45.0)	35 (52.2)	0.329
Age (years), Mean (SD)	6.85 ​± ​2.38	7.08 ​± ​2.49	6.35 ​± ​2.06	0.048
BMI (kg/m^2^), Mean (SD)	15.89 ​± ​2.55	15.85 ​± ​2.52	15.99 ​± ​2.65	0.553
Vaginal delivery, yes, n (%)	118 (57.0)	76 (54.3)	42 (62.7)	0.253
Preterm delivery, yes, n (%)	8 (3.9)	3 (2.1)	5 (7.5)	0.115
Parental education level, n (%)				0.216
High school or below	45 (21.7)	33 (23.6)	12 (17.9)	
Bachelor or junior college	138 (66.7)	88 (62.9)	50 (74.6)	
Master or above	24 (11.6)	19 (13.6)	5 (7.5)	
Feeding pattern, n (%)				0.814
Breast feeding	113 (54.6)	78 (55.7)	35 (52.2)	
Mixed feeding	74 (35.8)	48 (34.3)	26 (38.8)	
Formula feeding	20 (9.7)	14 (10.0)	6 (9.0)	
Passive smoke exposure, yes, n (%)	70 (33.8)	43 (30.7)	27 (40.3)	0.173
Renovation in the past year, yes, n (%)	58 (28.0)	38 (27.1)	20 (30.0)	0.685
Vitamin C supplement, yes, n (%)	21 (10.1)	14 (10.0)	7 (10.5)	0.920
Severe pneumonia, yes, n (%)	191 (92.3)	129 (92.1)	62 (92.5)	0.921

AR, allergic rhinitis; SD, standard deviation; BMI, body mass index.

### 3.2 Types of MPs identified in BALF samples of children with MPP

**Fig. 1:**
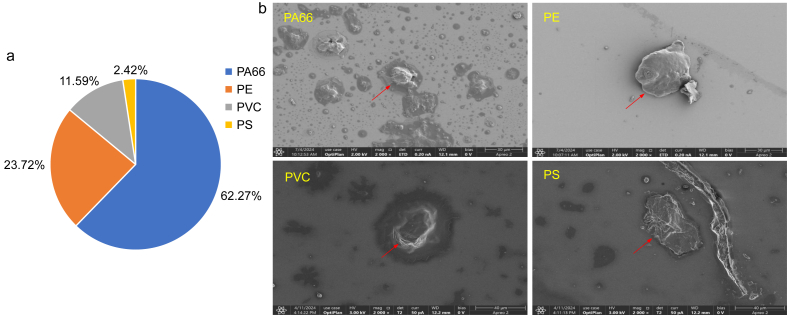
Characteristics of MPs concentrations, proportional composition, and morphology in BALF. (a) Average quality proportional composition of the top four measured MPs in BALF. (b) Representative images of PA66, PE, PVC, and PS in BALF captured using SEM.

Of the 11 ​MP types analyzed, 6 were detected. MPs were identified in 99.52% of samples. The detection rate of PA66 was the highest (92.27%), while PVC, PS, and PE were present in 80.68%, 78.74%, and 48.79% of BALF samples, respectively. Compared to the other studies [[31], [33]], PA66 and PVC had greater detection rates (Table 2). Among all detected MPs, PA66 was the most prevalent type (constituting 62.27%) (Fig. 1A). Furthermore, the geometric mean (GM) concentration of total MPs in each sample was 4.35 ​μg/mL BALF, while those of PA66, PE, PVC, and PS were 2.40, 0.87, 0.26, and 0.08 ​μg/mL BALF, respectively (Table 2). SEM imaging of representative MPs showed that their shapes were primarily fragmented (Fig. 1B). When MP concentrations were grouped by age, no significant differences were observed between the age groups (*P* ​> ​0.05) (Fig. S1).

**Table 2:** Bronchoalveolar lavage fluid (BALF) concentrations by different microplastics (MPs) (μg/mL) among 207 children.

MPs	The current study						Other studies					
	Detection rate (%)	GM	Overall, Median (IQR)	Non-AR, Median (IQR)	AR, Median (IQR)	*P* value	Artery, adults^b^ (n ​= ​17)			Marrow, adults^c^ (n ​= ​16)		
							Detection rate (%)	Accounting (%)	Abundance (μg/g)	Detection rate (%)	Accounting (%)	Abundance (μg/g)
Total MPs	99.52	4.35	3.86 (2.77, 6.20)	3.74 (2.63, 5.73)	4.49 (3.13, 7.36)	**0.045**	100.0	100.0	118.7	100.0	100.0	51.3
PA66	92.27	2.40	2.33 (1.57, 3.85)	2.09 (1.56, 3.57)	2.95 (1.65, 4.93)	**0.037**	58.8	15.5	31.3^a^	75.0	13.3	6.8^a^
PE	48.79	0.87	0.45 (0.45, 1.72)	0.45 (0.45, 1.76)	0.45 (0.45, 1.72)	0.838	17.6	1.1	7.2^a^	93.8	58.5	30.0^a^
PVC	80.68	0.26	0.38 (0.09, 0.62)	0.37 (0.11, 0.62)	0.40 (0.04, 0.68)	0.865	47.1	9.7	24.3^a^	75.0	33.2	17.0^a^
PS	78.74	0.08	0.08 (0.05, 0.12)	0.08 (0.05, 0.11)	0.08 (0.05, 0.13)	0.648	/	/	/	100.0	10.3	5.3^a^

GM, geometric mean; IQR, interquartile range; PA66, Polyamide 66; PE, Polyethylene; PVC, Polyvinyl Chloride; PS, Polystyrene.

a The mean abundance of each type of MPs is calculated by multiplying the abundance of total MPs by the proportion of each MP represented.

b Microplastics in three types of human arteries were detected by pyrolysis-gas chromatography/mass spectrometry (Py-GC/MS) [31].

c Discovery and analysis of microplastics in human bone marrow [33].

### 3.3 Association between exposure concentration of MPs and AR prevalence

Table 3 shows the associations of different types of MPs and total MPs with the presence of AR. In unadjusted Model 1, children with PA66 levels above the median had a significantly higher risk of AR (OR 1.81, 95% CI 1.00–3.27) compared to those below the median. After adjustment for confounders, the risk estimates increased (Model 2: OR 2.75, 95% CI 1.17–6.49; Model 3: OR 3.00, 95% CI 1.23–7.34). Although higher concentrations of total MPs, PE, PVC, and PS were also associated with elevated AR risk, these associations did not reach statistical significance. The corrected *P*-values using Bonferroni correction were compared with the uncorrected results.

**Table 3:** Logistic regression of the association between four microplastics (MPs) exposure concentrations and prevalence of allergic rhinitis (n ​= ​207).

MPs exposure, μg/mL	Model 1	Model 2	Model 3
Total MPs			
≤3.86	Reference	Reference	Reference
>3.86	1.51 (0.84, 2.72)	1.61 (0.71, 3.65)	1.65 (0.70, 3.92)
Log_10_ Total MPs	1.74 (1.02, 2.99)^a^	2.44 (1.17, 5.10)^a^	2.59 (1.20, 5.58)^a^
PA66			
≤2.33	Reference	Reference	Reference
>2.33	1.81 (1.00, 3.27)^a^	2.75 (1.17, 6.49)^a^	3.00 (1.23, 7.34)^a^
Log_10_ PA66	1.67 (1.07, 2.62)^a^	2.38 (1.27, 4.46)^a^	2.59 (1.34, 5.00)^a^
PE			
≤0.45	Reference	Reference	Reference
>0.45	1.03 (0.57, 1.84)	0.90 (0.39, 2.05)	0.92 (0.39, 2.16)
Log_10_ PE	1.08 (0.73, 1.60)	1.07 (0.61, 1.88)	1.10 (0.61, 1.98)
PVC			
≤0.38	Reference	Reference	Reference
>0.38	1.16 (0.65, 2.07)	1.37 (0.59, 3.14)	1.29 (0.55, 3.04)
Log_10_ PVC	0.96 (0.78, 1.19)	0.98 (0.73, 1.32)	0.94 (0.69, 1.28)
PS			
≤0.08	Reference	Reference	Reference
>0.08	1.06 (0.59, 1.90)	1.76 (0.77, 4.02)	1.64 (0.69, 3.89)
Log_10_ PS	1.12 (0.80, 1.57)	1.58 (1.00, 2.52)	1.53 (0.94, 2.48)

a *P* value ​< ​0.05. Model 1 was not adjusted. Model 2 was adjusted for age, sex, BMI, delivery mode, preterm delivery, and parental education level. Model 3 was further adjusted for feeding pattern, passive smoke exposure, home decoration in the past year, vitamin C supplementation, and severity of MPP.

### 3.4 Age-specific associations of MP concentrations with AR risk

**Fig. 2:**
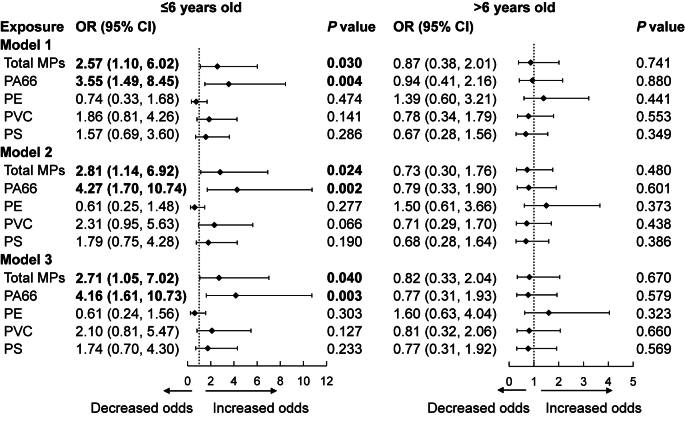
The association between microplastics (MPs) exposure and prevalence of allergic rhinitis (AR) by age group (n ​= ​207). The outcome is the odds ratio (OR) of AR for the MP exposure more than median level, with MP exposure less than median level serving as the control group. Model adjusted for age, sex, BMI, delivery mode, preterm delivery, parental education level, feeding pattern, passive smoke exposure, home decoration in the past year, vitamin C supplementation, and severity of MPP in Model 3.

As shown in Fig. 2, children aged ≤6 years with total MPs above the median had a significantly high risk of AR (OR [95% CI]: 2.57 [1.10–6.02] in Model 1; 2.81 [1.14–6.92] in Model 2; 2.71 [1.05–7.02] in Model 3). The association was even stronger for PA66 exposure (OR [95% CI]: 3.55 [1.49–8.45] in Model 1; 4.27 [1.70–10.74] in Model 2; 4.16 [1.61–10.73] in Model 3). In contrast, no association between AR and exposures to PS, PE, or PVC was observed in either age group.

Sensitivity analyses confirmed the robustness of the findings. Excluding pneumonia severity as a covariate (Model 4) produced comparable results to Model 3, indicating minimal confounding by severity. A multi-polymer model, further adjusting for co-occurring MPs (Model 5), also aligned with Model 3 (Table S6).

### 3.5 Dose-response relationship of total MPs and PA66 with AR risk

**Fig. 3:**
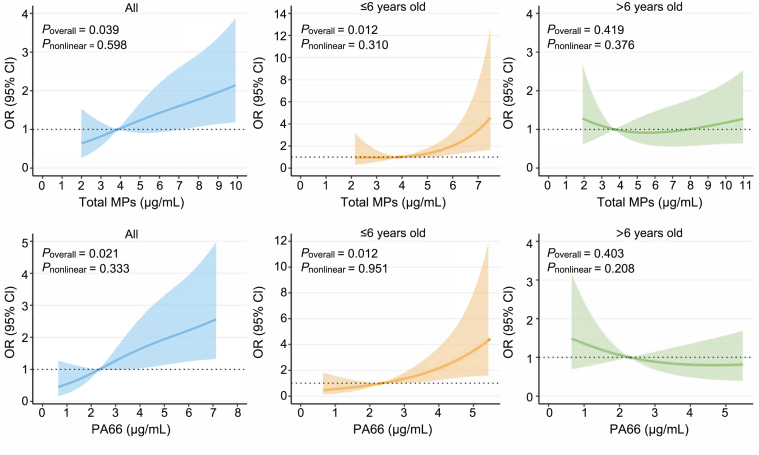
Dose-response relationships between total microplastics (MPs) and PA66 concentrations and prevalence of allergic rhinitis (AR) in children grouped by age (n ​= ​207). Model adjusted for age, sex, BMI, delivery mode, preterm delivery, parental education level, feeding pattern, passive smoke exposure, home decoration in the past year, vitamin C supplementation, and severity of MPP in Model 3.

Fig. 3 illustrates the dose–response relationships of total MPs and PA66 exposure with AR risk by age group. In all children, positive linear dose–response relationships were observed for both total MPs (*P*_overall_ ​= ​0.039, *P*_nonlinear_ ​= ​0.598) and PA66 (*P*_overall_ ​= ​0.021, *P*_nonlinear_ ​= ​0.333). Among children aged ≤6 years, these associations remained significant for total MPs (*P*_overall_ ​= ​0.012, *P*_nonlinear_ ​= ​0.310) and PA66 (*P*_overall_ ​= ​0.012, *P*_nonlinear_ ​= ​0.951). In contrast, no significant dose–response relationships were detected in children older than 6 years.

## 4 Discussion

This study observed a significant positive association between the exposure concentrations of total MPs and PA66 in pediatric BALF and the prevalence of AR, with evidence of a linear dose–response relationship. This association was particularly evident in children aged ≤6 years, whereas no significant dose–response pattern was observed in older children. To our knowledge, this represents the first epidemiological study linking MP exposure with AR in children.

MPs are found in many different settings and typically enter the human body through the air, food, and skin [34]. The respiratory system provides the greatest route of exposure, delivering an estimated (0.21–2.51) ​× ​10^6^ ​MP/year, approximately fivefold higher than dietary intake [5]. Since the skin serves as a basic barrier against environmental pollutants, the majority of MPs are unable to pass through it [35]. MPs <5 ​μm penetrate the alveoli, while those <2.5 ​μm deposit deep in lung tissue, potentially inducing oxidative stress and inflammatory injury [36]. The use of BALF as a biospecimen, therefore, provides a direct biomarker of exposure to the lower respiratory tract [37]. In this context, our analysis employed Py-GC/MS to quantify MPs with high sensitivity and demonstrated an almost universal detection (99.52%) in pediatric BALF. Detection rates exceeded those previously reported in adults, with PA66 (92.27%) as the predominant polymer, followed by PVC, PS, and PE—materials widely used in toys, textiles, furniture, and food-contact products [[38], [39], [40], [41], [42], [43]]. Children's behavioral patterns, such as floor-level play and hand-to-mouth activity, likely increase their risk of inhaling or ingesting MPs from these sources [44].

Notably, the concentrations we identified were markedly higher than those previously reported in BALF [6,45]. Uogintė et al. [45] found that the exposure to MPs in adult BALF samples from patients undergoing diagnostic bronchoscopy ranged from 0.14 to 12.8 MPs/100 ​mL, whereas Chen et al. [6] found an average of (43.1 ​± ​27.7) MPs/100 ​mL. Using a mass-to-particle conversion factor of 3.77 ​× ​10^−2^ μg/particle for micron-sized fragmented MPs [46], our measured concentration of 3.86 ​μg/mL BALF equates to approximately 10,239 MPs/100 ​mL. This represents values 800- and 145-fold higher than the values reported by Uogintė and Chen, respectively. Elevated burdens may reflect children's physiological vulnerability, supported by evidence that neonates and children excrete substantially higher MPs in feces and inhale comparatively greater amounts than adults [47,48]. However, methodological differences in particle detection may also contribute to this discrepancy. Conventional optical techniques (e.g., Raman spectroscopy, μFTIR) cannot detect particles <1 ​μm [2], and even advanced approaches like TEM/EDX have limited resolution (about 20 ​nm) for nanoplastics [49]. In contrast, our Py-GC/MS analysis enabled comprehensive, size-independent polymer quantification, capturing smaller particles that penetrate deeper lung regions [50]. Critically, sub-5 μm pollutants reach bronchioles, while <2.5 ​μm particles deposit in alveoli [51]. Py-GC/MS thus provides enhanced sensitivity for detecting lung-deposited MPs, particularly explaining the high concentrations in juvenile BALF samples where smaller, more bioavailable particles dominate [23]. It should be noted that the mass-to-particle conversion factor used above is based on the assumption of a single polymer density. However, actual samples contain various polymers (e.g., PE, PS, PA66) with different densities, causing about 10%–20% variation in particle number estimates. Additionally, the complex size and shape of MPs make precise quantification difficult. Thus, this factor only gives a rough estimate. Future studies should consider varying polymer densities and utilize improved models to estimate particle numbers more accurately. Although our QA/QC protocols demonstrated high recovery rates and low limits of detection in spiked controls, real-world BALF samples present unique challenges. The co-presence of multiple polymer types and complex biological matrices may competitively bind to extraction agents, potentially altering recovery efficiencies. Therefore, while our quantitative data reflect a robust analytical methodology, the absolute concentrations reported here should be interpreted with consideration of potential matrix effects. Given the limited BALF volume, sample allocation prioritized Py-GC/MS for quantitative polymer profiling; LD-IR/SEM was conducted on representative, untreated aliquots solely for qualitative morphological corroboration. Consequently, imaging outputs are not intended to support inference on particle number or size distributions and should be interpreted within this methodological context.

AR is a prevalent and severe allergic disease in children. Beyond pulmonary toxicity, MPs induce systemic immune-metabolic dysregulation: clinical evidence confirms their role in chronic vascular inflammation and intrinsic links to insulin resistance-driven metabolic dysfunction, collectively compromising immune competence and increasing susceptibility to acute respiratory disorders [23,24]. We also observed the associations between specific MP components (PA66, PS, PE, PVC) and the prevalence of AR. The biological plausibility of MPs contributing to AR is well supported. MPs promote oxidative stress, airway inflammation, and immune dysregulation, contributing to allergic sensitization [[23], [24], [25]]. PA66, in particular, has demonstrated pro-inflammatory properties in macrophages and induces cytokine release (IL-4, IL-5, IL-13, IFN-γ, TNF-α) in animal models [52,53]. Environmental co-exposures such as PS combined with DEHP aggravate immune skewing via the TRPA1–p38 MAPK axis, while MPs also disrupt gut microbiota and metabolites, fueling Th2-dominant airway inflammation through the gut–lung axis [21,22]. Our finding that PA66 exposure was associated with AR risk is consistent with both its ubiquitous use in children's environments and its experimental immunotoxicity. Studies on MPs and allergic diseases are still scarce. There is only one study that compared the nasal MP content of healthy individuals and AR patients. Although the content of various MP types was not examined, it was discovered that the nasal lavage fluid of 36 patients had a substantially higher MP abundance (3.10 pieces/mL) than that of the control group (2.38 pieces/mL) [16]. The findings of this study close a knowledge gap in the field by being the first to carefully investigate the association between various types of MP exposure and AR.

Our findings indicated an age-dependent susceptibility to the associations of MP concentrations on AR risk in children. This vulnerability may reflect developmental factors: 1) higher exposure due to floor-level activity, mouthing behavior, and use of plastic items [6,54]; and 2) immature airway barriers and immune systems, increasing sensitivity to environmental particles [55]. The dose–response patterns observed in younger children further support the association between MP exposure and AR risk among children, particularly among young children. However, confirmation will require further large-scale prospective cohort studies.

Strengths of our study include the use of highly specific polymer quantification by Py-GC/MS, adjustment for a wide range of confounders, and consistency of findings across sensitivity analyses. Furthermore, the age-stratified analysis provided novel insights into patterns of pediatric susceptibility.

Our study also has limitations. First, the modest sample size (n ​= ​207) limits precision, and inclusion was restricted to children with MPP, as BALF collection is clinically indicated only in this setting. Thus, our results are directly applicable to MPP-affected populations but may not be generalizable to all children. We attempted to minimize infection bias by adjusting for the severity of MPP, though residual confounding is possible. Second, the cross-sectional design precludes causal inference, and longitudinal studies are required to assess temporality. Third, only the four predominant MP types were analyzed, while other diverse polymers may also contribute. Finally, Py-GC/MS provides polymer mass rather than particle number or morphology, and complementary techniques (μFTIR, Raman) will be essential for future validation.

In conclusion, our findings indicate that MPs, particularly PA66, are significantly associated with increased AR risk in children, with the most pronounced associations observed in those under six years of age. These results underscore the urgent need to reduce MP exposure among vulnerable populations. Implementing public health interventions—such as tighter regulations on PA66 in products intended for direct contact with children—may help mitigate this risk. Future large-scale, community-based cohort and mechanistic studies are essential to clarify causality and uncover pathways through which MPs influence pediatric allergic disease.

## CRediT authorship contribution statement

**Huimin Li:** Writing – original draft, Methodology. **Jingli Yang:** Writing – review & editing, Methodology, Data curation, Conceptualization. **Lili Zhong:** Funding acquisition. **Gary W.K. Wong:** Writing – review & editing, Supervision, Conceptualization. **Han Huang:** Methodology. **Yinze Xu:** Writing – original draft, Methodology. **Wendi Ma:** Investigation, Data curation. **Xuelin Lv:** Writing – original draft. **Li Peng:** Methodology. **Dan Liu:** Methodology. **Niguang Xiao:** Methodology. **Shuhui Yin:** Methodology. **Qiong Wang:** Conceptualization. **Xiuqin Feng:** Investigation, Data curation. **Aimin Yang:** Writing – review & editing, Supervision, Conceptualization. **Jingjing Zhang:** Writing – review & editing, Supervision, Project administration, Funding acquisition, Conceptualization.

## Data availability

The datasets used and analyzed during the current study are available from the corresponding author on reasonable request.

## Declaration of competing interests

The authors declare that they have no competing financial interests or personal relationships.

## References

[1] Hartmann N.B., Hüffer T., Thompson R.C., Hassellöv M., Verschoor A., Daugaard A.E. (2019). Are we speaking the same language? Recommendations for a definition and categorization framework for plastic debris. Environ. Sci. Technol..

[2] Jenner L.C., Rotchell J.M., Bennett R.T., Cowen M., Tentzeris V., Sadofsky L.R. (2022). Detection of microplastics in human lung tissue using μFTIR spectroscopy. Sci. Total Environ..

[3] Dong X., Liu X., Hou Q., Wang Z. (2023). From natural environment to animal tissues: a review of microplastics(nanoplastics) translocation and hazards studies. Sci. Total Environ..

[4] Feng Y., Tu C., Li R., Wu D., Yang J., Xia Y. (2023). A systematic review of the impacts of exposure to micro- and nano-plastics on human tissue accumulation and health. Eco-Environ. Health.

[5] Yang Z., Wang M., Feng Z., Wang Z., Lv M., Chang J., Wang C. (2023). Human microplastics exposure and potential health risks to target organs by different routes: a review. Curr. Pollut. Rep..

[6] Chen C., Liu F., Quan S., Chen L., Shen A., Jiao A. (2023). Microplastics in the bronchoalveolar lavage fluid of Chinese children: associations with age, city development, and disease features. Environ. Sci. Technol..

[7] Borgatta M., Breider F. (2024). Inhalation of microplastics−a toxicological complexity. Toxics.

[8] Chen Y., Li X., Zhang X., Zhang Y., Gao W., Wang R. (2022). Air conditioner filters become sinks and sources of indoor microplastics fibers. Environ. Pollut..

[9] Ke D., Zheng J., Liu X., Xu X., Zhao L., Gu Y. (2023). Occurrence of microplastics and disturbance of gut microbiota: a pilot study of preschool children in Xiamen, China. EBioMedicine.

[10] Zhuge Y., Qian H., Zheng X., Huang C., Zhang Y., Li B. (2020). Effects of parental smoking and indoor tobacco smoke exposure on respiratory outcomes in children. Sci. Rep..

[11] Fan X.-L., Zeng Q.-X., Li X., Li C.-L., Xu Z.-B., Deng X.-Q. (2018). Induced pluripotent stem cell-derived mesenchymal stem cells activate quiescent T cells and elevate regulatory T cell response via NF-κB in allergic rhinitis patients. Stem Cell Res. Ther..

[12] Licari A., Magri P., De Silvestri A., Giannetti A., Indolfi C., Mori F. (2023). Epidemiology of allergic rhinitis in children: a systematic review and meta-analysis. J. Allergy Clin. Immunol. Pract..

[13] Frischmeyer-Guerrerio P.A., Guerrerio A.L., Oswald G., Chichester K., Myers L., Halushka M.K. (2013). TGFβ receptor mutations impose a strong predisposition for human allergic disease. Sci. Transl. Med..

[14] Acevedo N., Alashkar Alhamwe B., Caraballo L., Ding M., Ferrante A., Garn H. (2021). Perinatal and early-life nutrition, epigenetics, and allergy. Nutrients.

[15] Eguiluz-Gracia I., Mathioudakis A.G., Bartel S., Vijverberg S.J.H., Fuertes E., Comberiati P. (2020). The need for clean air: the way air pollution and climate change affect allergic rhinitis and asthma. Allergy.

[16] Tuna A., Taş B.M., Başaran Kankılıç G., Koçak F.M., Şencan Z., Cömert E. (2023). Detection of microplastics in patients with allergic rhinitis. Eur. Arch. Otorhinolaryngol..

[17] Krause S., Ouellet V., Allen D., Allen S., Moss K., Nel H.A., Manaseki-Holland S., Lynch I. (2024). The potential of micro- and nanoplastics to exacerbate the health impacts and global burden of non-communicable diseases. Cell Rep. Med..

[18] Prata J.C., da Costa J.P., Lopes I., Duarte A.C., Rocha-Santos T. (2020). Environmental exposure to microplastics: an overview on possible human health effects. Sci. Total Environ..

[19] Wu Q., Li R., You Y., Cheng W., Li Y., Feng Y. (2024). Lung microbiota participated in fibrous microplastics (MPs) aggravating OVA-induced asthma disease in mice. Food Chem. Toxicol..

[20] Lu K., Lai K.P., Stoeger T., Ji S., Lin Z., Lin X. (2021). Detrimental effects of microplastic exposure on normal and asthmatic pulmonary physiology. J. Hazard Mater..

[21] Lu S., Feng Q., Chen M., Zeng X., Wei H., Chen Q. (2024). Mechanisms underlying Th2-dominant pneumonia caused by plastic pollution derivatives (PPD): a molecular toxicology investigation that encompasses gut microbiomics and lung metabolomics. J. Hazard Mater..

[22] Han Q., Gao X., Wang S., Wei Z., Wang Y., Xu K. (2023). Co-exposure to polystyrene microplastics and di-(2-ethylhexyl) phthalate aggravates allergic asthma through the TRPA1-p38 MAPK pathway. Toxicol. Lett..

[23] Marfella R., Prattichizzo F., Sardu C., Fulgenzi G., Graciotti L., Spadoni T. (2024). Microplastics and nanoplastics in atheromas and cardiovascular events. N. Engl. J. Med..

[24] Dandona P., Aljada A., Bandyopadhyay A. (2004). Inflammation: the link between insulin resistance, obesity and diabetes. Trends Immunol..

[25] Wu H., Ballantyne C.M. (2020). Metabolic inflammation and insulin resistance in obesity. Circ. Res..

[26] National Health Commission of the People's Republic of China (2023). Guidelines for the diagnosis and treatment of Mycoplasma Pneumoniae Pneumonia in children (2023 edition). Int. J. Epidemiol. Infect. Dis..

[27] Boulet L.-P., Reddel H.K., Bateman E., Pedersen S., FitzGerald J.M., O'Byrne P.M. (2019). The global initiative for asthma (GINA): 25 years later. Eur. Respir. J..

[28] Brożek J.L., Bousquet J., Agache I., Agarwal A., Bachert C., Bosnic-Anticevich S. (2017). Allergic rhinitis and its impact on asthma (ARIA) guidelines-2016 revision. J. Allergy Clin. Immunol..

[29] von Elm E., Altman D.G., Egger M., Pocock S.J., Gøtzsche P.C., Vandenbroucke J.P. (2007). STROBE initiative, the strengthening the reporting of observational studies in epidemiology (STROBE) statement: guidelines for reporting observational studies. Lancet.

[30] Ma W., Zhong L., Yang J., Pinkerton K.E., Zhao S., Li H. (2025). Association between microplastic exposure and macrolide resistance in Mycoplasma pneumoniae pneumonia among younger children: a cross-sectional study in China. J. Hazard Mater..

[31] Liu S., Wang C., Yang Y., Du Z., Li L., Zhang M. (2024). Microplastics in three types of human arteries detected by pyrolysis-gas chromatography/mass spectrometry (Py-GC/MS). J. Hazard Mater..

[32] Lin Y.-C., Chen Y.-C., Kuo C.-H., Chang Y.-H., Huang H.-Y., Yeh W.-J. (2020). Antibiotic exposure and asthma development in children with allergic rhinitis. J. Microbiol. Immunol. Infect..

[33] Guo X., Wang L., Wang X., Li D., Wang H., Xu H. (2024). Discovery and analysis of microplastics in human bone marrow. J. Hazard Mater..

[34] Dang F., Wang Q., Huang Y., Wang Y., Xing B. (2022). Key knowledge gaps for one health approach to mitigate nanoplastic risks. Eco-Environ. Health.

[35] Li Y., Chen L., Zhou N., Chen Y., Ling Z., Xiang P. (2024). Microplastics in the human body: a comprehensive review of exposure, distribution, migration mechanisms, and toxicity. Sci. Total Environ..

[36] Akhbarizadeh R., Dobaradaran S., Amouei Torkmahalleh M., Saeedi R., Aibaghi R., Faraji Ghasemi F. (2021). Suspended fine particulate matter (PM_2.5_), microplastics (MPs), and polycyclic aromatic hydrocarbons (PAHs) in air: their possible relationships and health implications. Environ. Res..

[37] Che C., Li J., Dong F., Zhang C., Liu L., Sun X. (2020). Seasonal characteristic composition of inorganic elements and polycyclic aromatic hydrocarbons in atmospheric fine particulate matter and bronchoalveolar lavage fluid of COPD patients in Northeast China. Respir. Med..

[38] Liu S., Zheng J., Lan W., Yang Z., Li M., Li J. (2025). Microplastics exposed by respiratory tract and exacerbation of community-acquired pneumonia: the potential influences of respiratory microbiota and inflammatory factors. Environ. Int..

[39] (2024). Microplastics are everywhere - we need to understand how they affect human health. Nat. Med..

[40] Henry B., Laitala K., Klepp I.G. (2019). Microfibres from apparel and home textiles: prospects for including microplastics in environmental sustainability assessment. Sci. Total Environ..

[41] Zuo X. (2022). Development status and prospect of polyamide 66 industrial chain in China. China Synthetic Fiber Industry.

[42] Zhu X., Yao Y. (2022). Progress in preparation and application of superhydrophobic materials based on polyvinyl chloride. Chem. Ind. Eng. Prog..

[43] Miller R.R., Newhook R., Poole A. (1994). Styrene production, use, and human exposure. Crit. Rev. Toxicol..

[44] Mišľanová C., Valachovičová M., Slezáková Z. (2024). An overview of the possible exposure of infants to microplastics. Life.

[45] Uogintė I., Vailionytė A., Skapas M., Bolanos D., Bagurskienė E., Gruslys V. (2023). New evidence of the presence of micro- and nanoplastic particles in bronchioalveolar lavage samples of clinical trial subjects. Heliyon.

[46] Simon M., van Alst N., Vollertsen J. (2018). Quantification of microplastic mass and removal rates at wastewater treatment plants applying Focal Plane Array (FPA)-based Fourier Transform Infrared (FT-IR) imaging. Water Res..

[47] Zhang J., Wang L., Trasande L., Kannan K. (2021). Occurrence of polyethylene terephthalate and polycarbonate microplastics in infant and adult feces. Environ. Sci. Technol. Lett..

[48] Zuri G., Karanasiou A., Lacorte S. (2023). Microplastics: human exposure assessment through air, water, and food. Environ. Int..

[49] Moon S., Martin L.M.A., Kim S., Zhang Q., Zhang R., Xu W. (2024). Direct observation and identification of nanoplastics in ocean water. Sci. Adv..

[50] Qiu Q., Tan Z., Wang J., Peng J., Li M., Zhan Z. (2016). Extraction, enumeration and identification methods for monitoring microplastics in the environment. Estuar. Coast Shelf Sci..

[51] Fan Z., Bian Q. (2023). Research progress on the effects and mechanisms of airborne microplastics exposure on the respiratory system. Environ. Chem..

[52] Celebi Sozener Z., Ozdel Ozturk B., Cerci P., Turk M., Gorgulu Akin B., Akdis M. (2022). Epithelial barrier hypothesis: effect of the external exposome on the microbiome and epithelial barriers in allergic disease. Allergy.

[53] Busch M., Bredeck G., Waag F., Rahimi K., Ramachandran H., Bessel T. (2022). Assessing the NLRP3 inflammasome activating potential of a large panel of Micro- and nanoplastics in THP-1 cells. Biomolecules.

[54] Wu J., Lu A.D., Zhang L.P., Zuo Y.X., Jia Y.P. (2019). Study of clinical outcome and prognosis in pediatric core binding factor-acute myeloid leukemia. Zhonghua Xue Ye Xue Za Zhi.

[55] Brumberg H.L., Karr C.J. (2021). Council on environmental health, ambient air pollution: health hazards to children. Pediatrics.

