# Supplementary material for: Microplastic exposure in the lungs of young children and its associations with allergic rhinitis: A cross-sectional study in China
Source: Eco Environ Health. 2025 Oct 24;4(4):100193. doi: 10.1016/j.eehl.2025.100193 (PMC12766401; doi:10.1016/j.eehl.2025.100193)
Supplement: Multimedia component 1 [file mmc1.docx]

**Supplementary Information for**

**Microplastics exposure in the lungs of younger children and its associations with allergic rhinitis: A cross-sectional study in China**

Huimin Li^a, 1^, Jingli Yang^b, 1^, Lili Zhong^c, 1^, Gary W K Wong^d^, Han Huang^e^, Yinze Xu^a^, Wendi Ma^a^, Xuelin Lv^a^, Li Peng^c^, Dan Liu^c^, Niguang Xiao^c^, Shuhui Yin^c^, Qiong Wang^f^, Xiuqin Feng^c^, Aimin Yang^g, *^, Jingjing Zhang^a, *^

^*^Corresponding to: aiminyang@cuhk.edu.hk (A. Yang); zhangsmile1986@163.com (J. Zhang).

**Supporting Information**

Text S1: Sample and collection.

Text S2: Quality assurance and quality control (QA/QC)

Table S1. Py-GC/MS calibration curves of polymers.

Table S2. Characterization of microplastics in blank samples including procedure blank and sample blank

Table S3. Results of recovery test.

Table S4. The double range for microplastics exposure concentration (μg/mL).

Table S5. The number of allergic rhinitis and the samples size (%)

Table S6. The association between MPs exposure and AR risk in the sensitivity analysis

Figure S1. Age comparison of microplastics concentrations in the bronchoalveolar lavage fluid of children.

**Text S1: Sample and collection**

A senior physician performed the BALF sampling procedure uniformly by a senior physician. The diameter of the bronchoscope was selected based on the child's age, symptoms, intubation method, and anesthesia approach. A bronchoscope with a diameter ≤ 2.8 mm was used for neonates and infants, while a diameter of 4.0-4.9 mm was used for children above 1 year old refer to (refer to *Clinical Practice Guidelines for Bronchoalveolar Lavage in Chinese Children* [2024]). Sterile saline (0.9% w/v NaCl) was instilled for lavage, with the volume adjusted according to body weight: for children weighing <20 kg, the total lavage volume was 3 mL/kg, divided into three equal aliquots; for children weighing ≥20 kg, 20 mL was used per lavage, with a maximum volume of 3 mL/kg. For clinical testing purposes, 5-10 mL of fluid was collected per lavage, and any remaining BALF (<10 mL) was also collected. This process did not cause additional harm or economic burden to the participants. Samples were manually aspirated using a 20 mL plastic syringe, collected in glass bottles, and transported to the laboratory for storage at -80℃. Sterile saline pumped through the bronchoscope channel was obtained as blank controls and examined alongside BALF samples in order to track any possible sampling contamination. The MPs levels found in the BALF samples were subtracted from the background value of the blank control to determine the final MPs concentration.

**Text S2: Quality assurance and quality control (QA/QC)**

Throughout the entire experimental process, including sample collection, storage, processing, and analysis, strict protocols were followed to prevent contamination from environmental plastics and to ensure the precision of the outcomes. Disposable plastic suction catheters were employed during sample collection, and sample blanks were incorporated to account for any background values. To reduce the risk of microplastic contamination from the environment and procedures, several quality control measures were put into place throughout the sampling and laboratory stages: personnel were required to wear cotton lab coats and polymer-free nitrile gloves; the bronchoscopy and lavage rooms were maintained to the cleanliness standards of an operating room; and the laboratory was kept clear of any instruments or consumables not essential to the study. An experimental platform was shielded with aluminum foil to minimize the chance of environmental microplastic contamination. External standards were incorporated into each sample, and additional quality control samples, sample blanks, and procedural blanks were introduced at a rate of one per every 20 samples. Moreover, spiked samples and parallel samples were utilized to guarantee the precision and consistency of microplastic detection in BALF.

In order to evaluate the recovery performance of microplastic detection using Py-GC/MS, 11 standard substances were utilized to determine recovery rates. These substances included polyamide-6, polyamide-66, polycarbonate, polyethylene, polyethylene terephthalate, polymethyl methacrylate, polypropylene, polystyrene, polyvinyl chloride, polylactic acid, and Poly (butylene adipate-co-terephthalate). The extraction efficiency and recovery rates were assessed using the aforementioned standard solutions. Three BALF subsamples were spiked with the 11 microplastic standards and then subjected to the same pretreatment process as the samples prior to analysis. The recovery rate was calculated using the formula provided: C0 (μg/L) denotes the concentration of microplastics in the control BALF sample without added standards, while C2 (μg/L) is the concentration of microplastics detected in the BALF sample after the introduction of a known concentration C1 (μg/L) of standards. The limit of detection (LOD) and quantification (LOQ) were set at three and ten times the baseline noise, respectively.

$$\mathrm{recovery}\left( \% \right)=\frac{C2-C0}{C1} \times100\%$$

**Table S1. Py-GC/MS calibration curves of polymers**

| **Polymer** | **Characteristic compound** | **Retention time (min)** | **Quantifier ion**  **(*m/z*)** | **Calibration curve equation** | **R^2^** | **LOD**  **(μg)** | **LOQ**  **(μg)** |
| --- | --- | --- | --- | --- | --- | --- | --- |
| PA6 | ε-caprolactam | 8.9 | 85 113 | y=81.48x-2.1289 | 0.9986 | 0.02 | 0.06 |
| PA66 | cyclopentanone | 4.3 | 55 84 | y=4.3501x-0.9096 | 0.9945 | 0.02 | 0.06 |
| PC | bisphenol A | 13.8 | 213 228 | y=45.041x-2.1509 | 0.9923 | 0.02 | 0.06 |
| PE | n-monoene | 6.5 | 97 111 140 | y=10.933x-0.1179 | 0.9966 | 0.5 | 1.5 |
| PET | benzoic acid | 7.8 | 51 77 105 | y=7.4932x-19.327 | 0.9933 | 0.02 | 0.06 |
| PMMA | methyl methacrylate | 3.4 | 69 100 | y=367.04x-11.061 | 0.9983 | 0.02 | 0.06 |
| PP | 2,4-dimethyl-1-heptene | 4.9 | 70 126 | y=160.86x-1.2887 | 0.9905 | 0.05 | 0.15 |
| PS | styrene dimer | 15.5 | 51 78 104 | y=248.16x-3.1565 | 0.9925 | 0.02 | 0.06 |
| PVC | naphthalene | 8.1 | 115 128 130 | y=51.002x-3.7985 | 0.9907 | 0.02 | 0.06 |

Abbreviations: LOD, limit of detection; LOQ, limit of quantitation; PA, polyamide; PC, polycarbonate; PE, polyethylene; PET, polyethylene terephthalate; PMMA, polymethyl methacrylate; PP, polypropylene; PS, polystyrene; PVC, polyvinyl chloride; Py-GC/MS, pyrolysis-gas chromatography/mass spectrometry.

**Table S2. Characterization of microplastics in blank samples including procedure blank and sample blank**

| **Categories** | **Types** | **Microplastics concentration (μg/mL)** |
| --- | --- | --- |
| Procedure blank | Polyethylene (PE) | 0.04755 |
| Sample blank | Polystyrene (PS) | 0.2656 |
|  | Polymethyl methacrylate (PMMA) | 0.14531 |

**Table S3. Results of recovery test.**

| **Polymers** | **Abbreviation** | **Recovery (%)** |
| --- | --- | --- |
| Polyamide 6 | PA6 | 102.03 |
| Polyamide 66 | PA66 | 107.33 |
| Polyethylene | PE | 105.20 |
| Polyvinyl chloride | PVC | 83.99 |
| Polystyrene | PS | 93.80 |
| Polypropylene | PP | 106.47 |
| Polymethyl methacrylate | PMMA | 91.75 |
| Polyethylene terephthalate | PET | 81.99 |
| Polycarbonate | PC | 98.87 |

**Table S4. The double range for microplastics exposure concentration (μg/mL).**

| **Double** | **N** | **Total MPs** | **PA66** | **PE** | **PVC** | **PS** |
| --- | --- | --- | --- | --- | --- | --- |
| D1 | 103 | ≤ 3.86 | ≤ 2.33 | ≤ 0.45 | ≤ 0.38 | ≤ 0.08 |
| D2 | 104 | > 3.86 | > 2.33 | > 0.45 | > 0.38 | > 0.08 |

Notes: D1 and D2 represent dichotomized groups based on the median concentration of each microplastic type. Threshold values are derived from the calculated median of the entire cohort (N=207). D1: Low-exposure group (concentration ≤ median); D2: High-exposure group (concentration > median)

Abbreviations: Total MPs, Total microplastics; PA, polyamide; PE, polyethylene; PVC, polyvinyl chloride; PS, polystyrene.

**Table S5. The number of allergic rhinitis and the samples size (%)**

| **MPs exposure, μg/mL** | ≤ **Median levels** | **> Median levels** |
| --- | --- | --- |
| Total MPs |  |  |
| Overall | 29/104(27.9) | 38/103(36.9) |
| ≤ 6 years old | 12/48(25) | 24/52(46.2) |
| > 6 years old | 17/56(30.4) | 14/51(27.5) |
| PA66 |  |  |
| Overall | 27/104(26) | 40/103(38.8) |
| ≤ 6 years old | 11/50(22) | 25/50(50) |
| > 6 years old | 16/54(29.6) | 15/53(28.3) |
| PE |  |  |
| Overall | 34/106(32.1) | 33/101(32.7) |
| ≤ 6 years old | 19/48(39.6) | 17/52(32.7) |
| > 6 years old | 15/58(25.9) | 16/49(32.7) |
| PVC |  |  |
| Overall | 32/104(30.8) | 35/103(34) |
| ≤ 6 years old | 17/57(29.8) | 19/43(44.2) |
| > 6 years old | 15/47(31.9) | 16/60(26.7) |
| PS |  |  |
| Overall | 33/104(31.7) | 34/103(33) |
| ≤ 6 years old | 14/46(30.4) | 22/54(40.7) |
| > 6 years old | 19/58(32.8) | 12/49(24.5) |

Abbreviations: MPs, microplastics; PA66, Polyamide 66; PE, Polyethylene; PVC, Polyvinyl Chloride; PS, Polystyrene.

**Table S6. The association between MPs exposure and AR risk in the sensitivity analysis**

| **MPs exposure, μg/mL** | **Model 4** | **Model 5** |
| --- | --- | --- |
| Total MPs |  |  |
| Overall | 1.64 (0.69,3.87) | -- |
| ≤ 6 years old | **2.78 (1.08,7.18)** | -- |
| > 6 years old | 0.81 (0.33,2.02) | -- |
| PA66 |  |  |
| Overall | **2.95 (1.21,7.17)** | **2.86 (1.11,7.36)** |
| ≤ 6 years old | **4.19 (1.62,10.79)** | **4.13 (1.50,11.40)** |
| > 6 years old | 0.74 (0.30,1.86) | 0.78 (0.28,2.17) |
| PE |  |  |
| Overall | 0.92 (0.39,2.16) | 1.25 (0.49,3.21) |
| ≤ 6 years old | 0.62 (0.24,1.58) | 1.24 (0.45,3.41) |
| > 6 years old | 1.65 (0.66,4.16) | 0.82 (0.29,2.26) |
| PVC |  |  |
| Overall | 1.28 (0.54,3.02) | 0.84 (0.34,2.08) |
| ≤ 6 years old | 2.16 (0.84,5.58) | 0.51 (0.18,1.46) |
| > 6 years old | 0.79 (0.31,2.02) | 1.72 (0.68,4.35) |
| PS |  |  |
| Overall | 1.62 (0.69,3.83) | 0.92 (0.36,2.32) |
| ≤ 6 years old | 1.81 (0.73,4.46) | 1.53 (0.54,4.35) |
| > 6 years old | 0.74 (0.30,1.83) | 0.91 (0.33,2.53) |

Notes: The outcome is the odds ratio (OR) of AR for the MPs exposure more than median level, with MPs exposure less than median level serving as the control group. Model 4 adjusted for age, sex, BMI, delivery mode, preterm delivery, parental education level, feeding pattern, passive smoke exposure, home decoration in the past year, and vitamin C supplementation; Model 5 was further adjusted for other type of microplastics.

Since Total MPs is composed of various other types of microplastics, in order to avoid excessive adjustment, multiple microplastic exposure adjustments were not made in Model 5 for Total MPs.


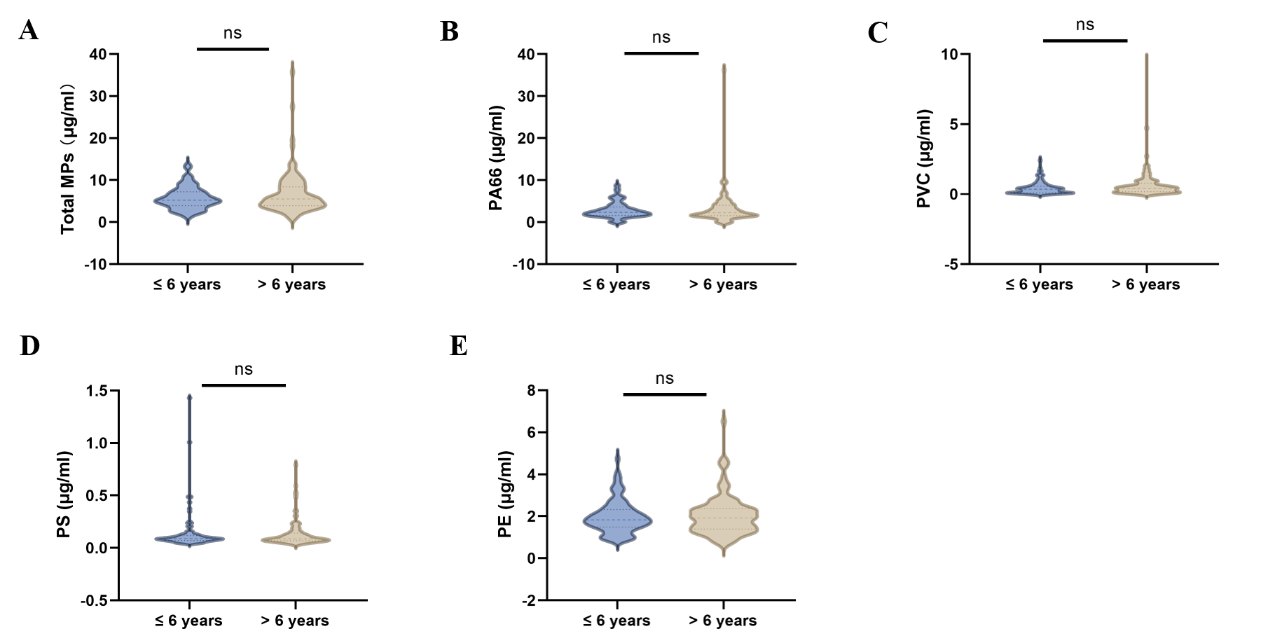


**Figure S1.** **Microplastics concentrations in the bronchoalveolar lavage fluid grouped by age**

A. Total MPs, Total microplastics; B. PA66, polyamide 66; C. PVC, polyvinyl chloride; D. PS, polystyrene; E. PE, polyethylene.
